# “Inequalities in access to medicines for diabetes and hypertension across the capitals in different regions of Brazil: a population-based study”

**DOI:** 10.1186/s12889-021-11279-6

**Published:** 2021-06-28

**Authors:** Vanessa Iribarrem Avena Miranda, Antônio Augusto Schäfer, Cristiane Damiani Tomasi, Jacks Soratto, Fernanda de Oliveira Meller, Marysabel Pinto Telis Silveira

**Affiliations:** 1 Postgraduate Program in Public Health, University of Southern Santa Catarina, Av. Universitária, 1105, Criciúma, Santa Catarina CEP: 88806-000 Brazil; 2 Postgraduate Program in Public Health, University of Southern Santa Catarina, Criciúma, Santa Catarina Brazil; 3 Postgraduate Program in Epidemiology, University Federal of Pelotas, Pelotas, Rio Grande do Sul Brazil

**Keywords:** Pharmaceutical services, Pharmacoepidemiology, Health inequalities, Cross-sectional studies, Diabetes, Hypertension

## Abstract

**Background:**

To guarantee prevention and adequate treatment, as required for the population to have access to health services and technologies, including medicines. The purpose of this study is to analyse the economic and regional inequalities in access to medicines for diabetes and hypertension among the adult population in Brazil.

**Methods:**

This was a cross-sectional study with adults aged 18 and over from the VIGITEL study conducted in 2019 in all Brazilian regions. Non-access to antidiabetic and antihypertensive drugs was assessed according to formal education and housing macro-region. The slope index of inequality (SII) was used to analyse absolute inequalities.

**Results:**

The total number of individuals interviewed was 52,443. Approximately 10.0% of the people with diabetes and/or hypertension reported not having access to drug treatment. The major means for having access to antihypertensive drugs, in all regions, was private pharmacies; for antidiabetics, in the North region, people had greater access through private pharmacies, while in the Northeast, Southeast and South, they had greater access through the public sector. Inequalities were found in the lack of access to medicines according to the region of residence, especially in the North region.

**Conclusion:**

The lack of access to medicines showed regional disparities, particularly in the most economically vulnerable regions.

## Background

Brazil continues in a growing process of epidemiological transition, with an increase in the life expectancy of the population in recent decades, and a complex scenario of overlapping health problems. This situation is aggravated by the emergence of communicable diseases and an increase in chronic noncommunicable diseases (NCD) [1, 2], especially affecting more vulnerable groups, with a low level of education and lower income [3].

In 2015, the United Nations (UN) set 17 Sustainable Development Goals (SDGs) to be achieved by 2030; one of them is to reduce premature NCD mortality by one third through adequate prevention and treatment [4]. Model data predictors of mortality for the period 2000 to 2033 indicate that, among diseases of the circulatory system, diabetes and hypertension will continue to rank among the major health problems [5], and they will cause increasing demand for the need to provide access to diagnosis and adequate treatment for the population [6].

In low- and middle-income countries, adults face almost twice as much risk of death from NCDs than those from high-income countries [5]. In Brazil, in 2013, 72.6% of deaths were due to NCDs, and cardiovascular diseases were the most frequent, accounting for 29.7% of deaths, followed by neoplasms (16.8%), chronic respiratory diseases (5.9%) and diabetes (5.1%). These four diseases accounted for 85% of deaths caused by CNCD [6].

To guarantee prevention and adequate treatment, the population needs to have access to health services and technologies, including medicines. Access to essential medicines for all individuals is not only a priority in current health policies, but also a fundamental right that has been widely recognized worldwide [7, 8].

Given the importance of universal access to medicines for the control of NCDs, as well as the identification of disparities in access to this treatment, the aim of this article is to analyse the economic and regional inequalities in access to medicines for diabetic and hypertensive patients among the adult population of Brazil.

## Methods

A cross-sectional population-based study was carried out with Brazilian adults aged 18 years or older. Data were collected from the Surveillance of Risk and Protection Factors for Chronic Diseases by Telephone Survey (VIGITEL) survey, conducted in 2019. VIGITEL is held annually in all Brazilian capitals and the Federal District and aims to monitor the frequency and the distribution of the main determinants of chronic noncommunicable diseases (NCDs) [9].

For the selection of individuals, probabilistic samples were taken of adults living in households with at least one landline. For the calculation of risk factor estimates, a 95% confidence coefficient and a maximum error of 2 pp. were considered. For specific estimates, according to sex, 3 pp. of maximum error was considered. The sample selection was carried out in two stages. In the first one, at least 5000 telephone lines per city were selected systematically and stratified by postal code (CEP). Afterwards, the lines were drawn and divided into replicas of 200 lines, which had the same proportion of lines per CEP as the original register.

Initially, 197,600 telephone lines were selected, and to reach the minimum number of 2000 interviews per capital, 36 replicates were used per city, with a range of 30 to 56 replicates, depending on the state. In the second stage, one of the eligible adults residing in the selected household was selected. More details about the research can be found in a previously published report [9].

The following lines were not eligible for the survey: they were business telephone lines; they no longer existed or were out of service; in addition, the lines that did not respond to six attempts at calls made on different days and times, including Saturdays and Sundays and night periods, and which probably corresponded to closed households.

The applied questions addressed sociodemographic characteristics and information on health status and various risk factors for NCDs. The dependent variables analysed in this study were the report of diabetes or hypertension previously diagnosed by a doctor, the use of drugs (for those who had these diseases), the source for obtaining the drugs, and lack of access.

Information on the use of medications was collected through the following questions: “Are you currently taking any medications to control high blood pressure?”, “In the past 30 days, have you been left without any medications to control high blood pressure for some time?” and “How do you get your medication for high blood pressure?”, with the following response options: Private pharmacy (direct purchase), Brazilian Unified Health System (SUS) – (government health facilities) and People’s Pharmacy Program (PPP). The PPP was launched in 2004 by the federal government. This strategy aims to promote the expansion of access to medication to the entire population. The purpose is to avoid withdrawal of treatment, especially by individuals with low income who use private health services, but have difficulty in buying the required medications in regular pharmacies [10, 11]. The same questions were asked about diabetes medications.

The independent variables were age in full years, according to four categories (18–24; 25–39; 40–59; ≥60), sex (male; female), self-reported skin color (white; black; brown), education in years (none; 1–4; 5–8; 9–11; ≥12) and region of residence (North; Northeast; Midwest; Southeast; South).

Data analysis was performed using the STATA® statistical program, version 15.0. The weightings related to the complex sample design were considered using the Rake method [12], which corrects the estimates and provides reliable information for the adult population with landline in each municipality. The reason is that the use of this weight equates the sociodemographic composition estimated from the VIGITEL sample in each town or city to the sociodemographic composition estimated for the total adult population of the same town or city.

The effect of sample design was considered for all analyses, using the set of *svy* commands, specific for the analysis of surveys based on complex samples of the statistical program Stata 15.0. The sample was described for the independent variables, and the prevalence of outcomes (hypertension and diabetes) was calculated with the respective confidence intervals using Pearson’s Chi-square test, using the significance level of 0.05.

**Fig. 1:**
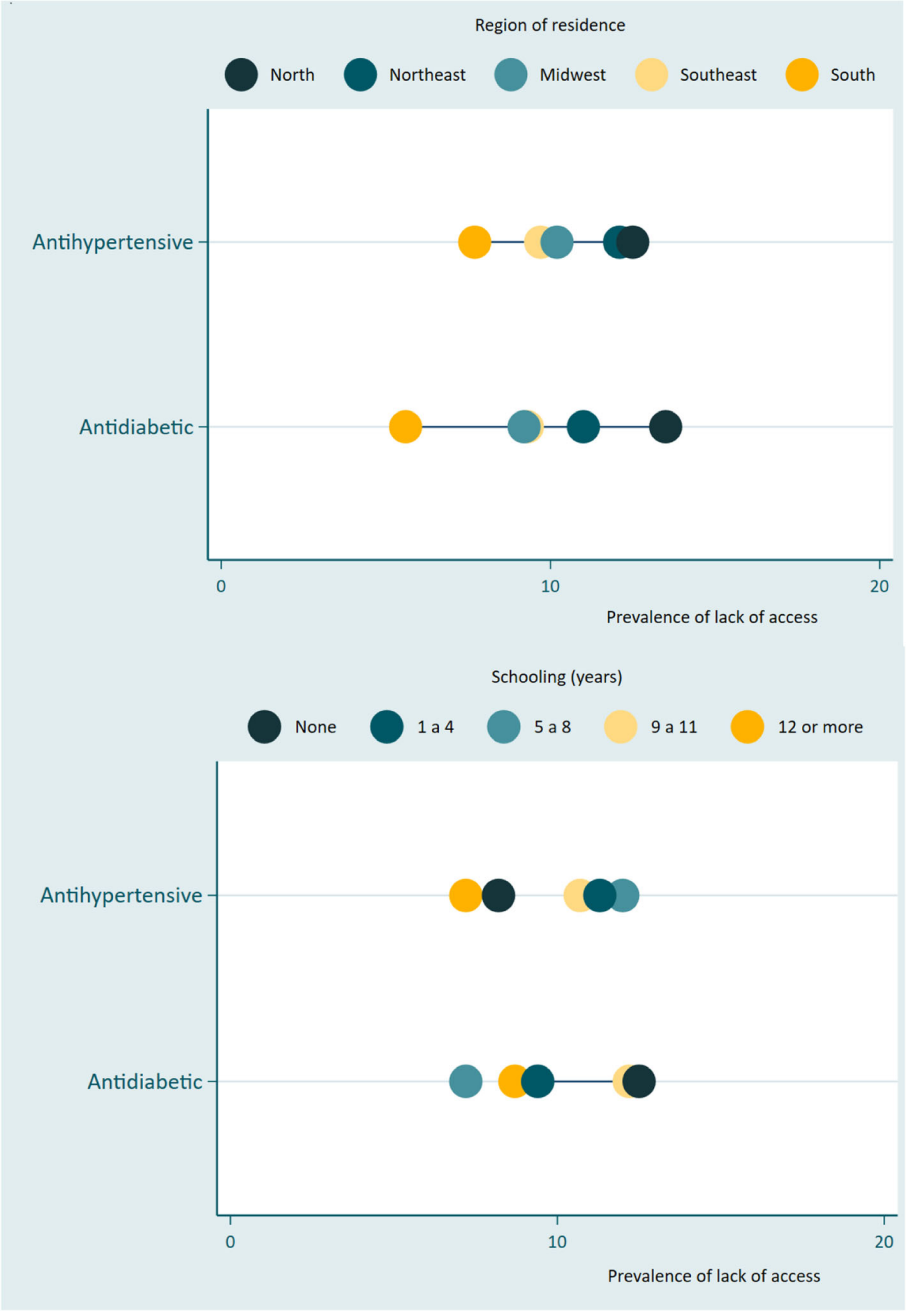
Inequalities in the lack of access to medicines for hypertension and diabetes among adults over 18 years old according to Region of residence and formal education Vigitel. 2019

Formal analyses of economic and regional inequalities in access to medicines for diabetic and hypertensive patients were carried out considering, respectively, formal education and macro-region of residence of the individuals. To identify possible inequalities, the slope index (SII) was used [13]. The SII is a measure of absolute inequality, based on the difference in the values of a given outcome between the extremes of the distribution, through a logistic regression for binary outcomes. It is expressed in percentage points ranging from 100 and 100, where zero represents no inequality, and negative values are translated as the poorest group having high prevalence of the outcome [13]. A significance level of 0.05 was considered. To better illustrate these differences between the subgroups, equiplot graphics were used (Fig. 1).

The VIGITEL research project was approved by the National Commission for Ethics in Research for Human Beings of the Ministry of Health (CAAE: 65610017.1.0000.0008). A Free and Informed Consent was obtained verbally at the time of the telephone call that was carried out by a central office and had all the interviews recorded for the purpose of data quality control.

## Results

Of the total sample of 52,443 individuals, 7.5% (95% CI 7.0; 7.8) reported having diabetes; of these, 84.0% were using oral diabetes medications, and 20.4% were using insulin. Of the 24.5% (95% CI 23.8; 25.3) who reported having hypertension, 83.4% reported being on medication. Total access to medicines was 90% of the population. On the other hand, approximately 10.0% of people with diabetes and/or hypertension reported a lack of access to these drugs.

Table 1 shows the description of the interviewed sample and the prevalence of lack of access to medicines for diabetes and hypertension according to sociodemographic characteristics. It was found that the lack of access decreases with increasing age for both drugs. Furthermore, the lack of access to antihypertensive drugs was greater in those individuals with less education (1–4 and 9–11 years).

**Table 1:** Sample characteristics and prevalence of lack of access to medicines for diabetes and hypertension

Variable	Total sample	Lack of access to diabetes medications			Lack of access to medicines for hypertension		
	%	%	95% CI	*P* value	%	95% CI	*P* value
**Gender**				0.177			0.601
Male	46.0	11.0	8.3; 14.3		10.0	8.0; 12.3	
Female	54.0	8.7	7.1; 10.7		10.6	9.4; 11.9	
**Age (years)**				0.025^a^			< 0.001^a^
18–24	13.7	19.3	6.8; 43.9		17.7	5.9; 42.3	
25–39	33.4	16.1	9.3; 26.2		12.3	8.7; 17.0	
40–59	34.6	10.5	7.8; 13.9		12.8	10.8; 15.2	
60 or more	18.3	7.8	6.2; 9.7		7.6	6.7; 8.6	
**Ethnicity**				0.115			0.357
White	43.8	8.7	6.4; 11.6		9.7	8.1; 11.7	
Black	11.4	15.2	9.6; 23.3		9.9	7.4; 13.0	
Brown	44.8	10.3	7.9; 13.4		11.4	9.7; 13.4	
**Education (years)**				0.279			0.037
none	2.1	12.5	5.1; 27.3		8.1	5.7; 11.5	
1–4	10.7	9.4	6.8; 12.8		11.3	8.7; 14.5	
5–8	16.0	7.2	4.9; 10.3		12.0	9.6; 14.7	
9–11	38.4	12.2	9.1; 16.1		10.7	8.9; 12.6	
12 or more	32.8	8.7	6.0; 12.4		7.2	5.8; 9.0	
**Region of residence**				0.188			0.060
North	10.4	13.4	9.6; 18.3		12.5	10.2; 15.3	
Northeast	25.2	11.0	9.0; 13.3		12.1	10.7; 13.5	
Midwest	11.8	9.2	5.3; 15.4		10.2	7.9; 13.1	
Southeast	44.6	9.3	6.7; 12.6		9.7	7.8; 11.9	
South	8.0	5.6	3.5; 8.6		7.7	5.9; 9.9	

*P*-value: chi-square test for heterogeneity

^a^ *p*-value of the linear trend test

Regarding the characteristics of individuals according to chronic diseases, there was a higher prevalence of hypertension among female individuals, with a positive trend with age. On the other hand, the prevalence tended to decrease with the increase in schooling. The region with the highest prevalence of hypertensive patients was the Midwest, followed by the Southeast. Regarding individuals with diabetes, there were no differences between males and females, and there was a trend of increasing prevalence with advancing age. For the education variable, there was an inversely proportional trend. In addition, there were differences in the prevalence of diabetes among regions, with the Southeast being the one with the highest proportion (data not shown in the table).

When assessing the main source of access to medicines according to the three study sources (SUS, Popular pharmacies or Private pharmacies), the main means of obtaining antihypertensive drugs in all regions was private pharmacies, whereas for antidiabetics, the main source of obtaining varied according the region of the country, with a predominance of SUS in all regions except for the Midwest.

Table 2 and Fig. 1 shows the inequalities in the lack of access to medicines for hypertension and diabetes among adults over 18 years old according to Region of residence and formal education.

**Table 2:** Absolute inequality (Slope Index-SII) in the lack of access to medicines for hypertension and diabetes in relation to the education and region of residence of the studied Brazilians. Vigitel. 2019

	**Prevalence of lack of access according to schooling**							
	**none**	**1 a 4**	**5 a 8**	**9 a 11**	**12 or more**	***P*** **value**^**a**^	**SII**	***P*** **value**^**b**^
Antihypertensive	8.2	11.3	12.0	10.7	7.2	0.037	−3.6	0.056
Antidiabetic	12.5	9.4	7.2	12.2	8.7	0.279	0.7	0.825
	**Prevalence of lack of access by region of residence**							
	**North**	**Northeast**	**Midwest**	**Southeast**	**South**	***P*** **value**^**a**^	**SII**	***P*** **value**^**b**^
Antihypertensive	12.5	12.1	10.2	9.7	7.7	0.065	−4.7	0.004
Antidiabetic	13.5	11.0	9.2	9.3	5.6	0.188	−5.4	0.020

^a^ *p*-value of the Chi-square test for linear trend

^b^ *p* value from Wald test

**Fig. 2:**
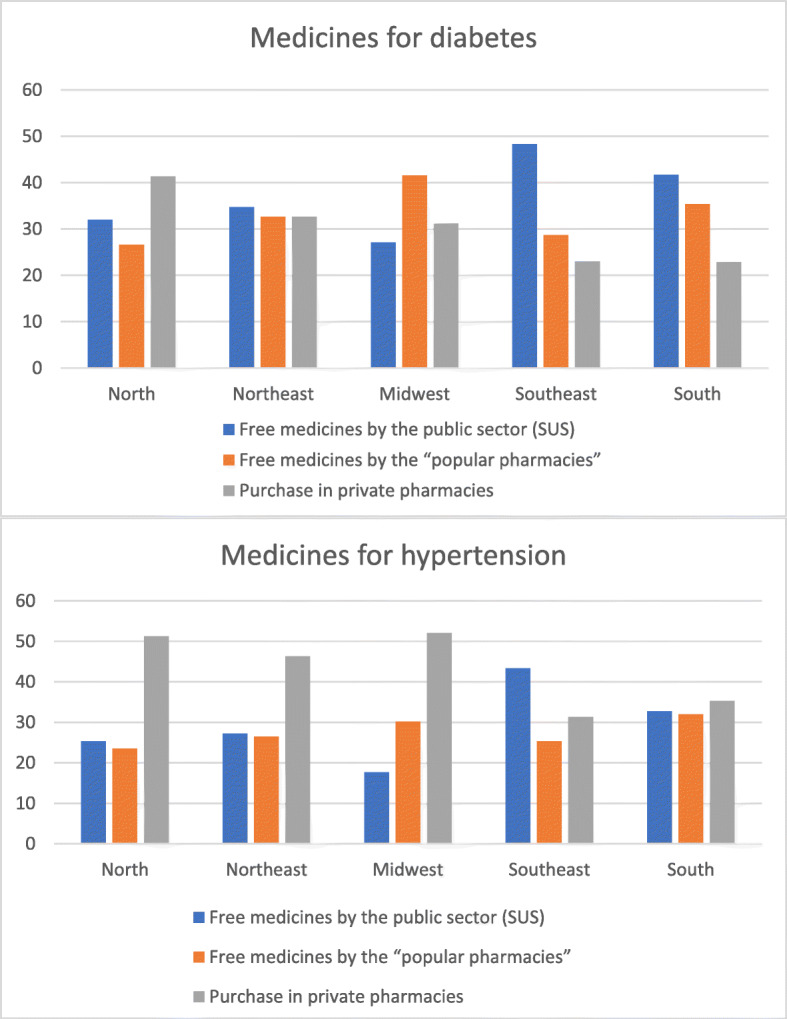
Source of access to medicines for hypertension and diabetes according to the region of residence of the Brazilians studied. Vigitel. 2019

Regarding the source of access to medicines for hypertension and diabetes according to the region of residence, it was found that the main means of obtaining antihypertensive drugs in all regions was through purchase in private pharmacies; the rate was higher in the Midwest (52.1%) and North (51.2%) regions. For antidiabetic drugs, no standard of achievement was found. The North region had greater access through private pharmacies (41.3%), while the Northeast, Southeast and South regions had greater access through SUS (34.7, 48.3 and 41.7%, respectively) (Fig. 2).

According to the inequality index, inequalities were found when assessing the lack of access to medicines according to region of residence. The result indicates that the lack of access to anti-hypertensive and anti-diabetic drugs occurs in greater magnitude in the residents of the North region. For medicines for hypertension and diabetes, residents in the Northeast have less access than residents in the South (Table 2).

About formal education, the indices suggest that individuals with a low level of education have, on average, less access to antihypertensive drugs when compared to those with a higher level of education; however, the estimates were not significant.

## Discussion

Access to medicines is part of the right to health, which in turn must be promoted through the adoption of public policies and, in some cases, legislative mechanisms to ensure them [14, 15]. Nevertheless, there is an inequity in this guarantee, which reinforces the need to strengthen the Unified Health System for free supply of medicines with a view to reducing inequalities [16].

Among the respondents, 84.0% of diabetic patients and 83.4% of hypertensive patients were using drug treatment. It is known that the control of both chronic diseases is based on a series of precautions, which involve nutritional changes, physical activity and control of some risk factors [17, 18]. However, drug treatment becomes the most effective way to control and prevent complications of these morbidities, as adherence to lifestyle changes is always lower than adherence to treatment [19]. However, for this treatment to be effective, users must have access to it [20].

A study conducted with data from the 2011 VIGITEL survey showed a lower prevalence of medication use among diabetic and hypertensive patients, with 78.2 and 71.0%, respectively, compared to findings of the present article [21]. The National Health Survey (2013) showed that 80.2 and 81.4% used drugs to control diabetes and hypertension, respectively [22]. These results suggest a progression in the use of medications for these two chronic conditions. The increase in the use of medicines for these diseases may also reflect a less healthy behavior, which leads to an increase in the prevalence of hypertension and diabetes, and increases the number of people who need these medicines. However, data from the PNAUM study (21) [23], collected between September 2013 and February 2014, indicated greater access to medicines to treat hypertension (94.6%); when compared by region, such access was higher in the South and lower in the Midwest and Northeast, confirming the regional inequality found in this analysis, as for both hypertension and diabetes, access was greater in the South and lesser in the North and Northeast.

The general lack of access to medicines for diabetes and/or hypertension was approximately 10.0%. Although the findings indicate that the constitutional right of health may be compromised by a portion of the investigated participants, it should be noted that there is a considerable level of access to antihypertensives and antidiabetics in Brazil, and this is due to a series of public policies that have been adopted to guarantee universal and free access to medicines [24].

In 1998, Brazil instituted the National Medicines Policy [8] and adopted, among other guidelines, the National List of Essential Medicines [14]. Subsequently, the generic medicine policy was also implemented, whose objective was to expand access to medicines with guaranteed quality and at a more affordable price for the population. In 2004, the Popular Pharmacy Program (PFP) was created within the scope of the System Unified Health System (SUS) [25]. PFP emerged with the aim of expanding access to medicines to the entire population, aiming to prevent withdrawal of treatment, especially in low-income individuals who cannot afford to buy the medicines they need in private pharmacies [26]. In 2011, the program was redesigned to further increase the coverage of access to medicines and promote comprehensive health care, changing its name to “Health is priceless”, in which medicines for the treatment of diabetes, hypertension and asthma began to be provided free of charge [11].

After a separate analysis of the three sources of access to medicines, it was found that the main means of obtaining antihypertensive drugs in the North, Northeast and Midwest regions was the private pharmacy. This result is similar to the one reported in the VIGITEL 2011 study [21]. This finding may reflect a series of barriers that still exist, e.g., difficulty in scheduling a medical consultation to renew the prescription to be to obtain the medications, lack of knowledge about the list of medications available for free, prejudice against free medications provided by the government, and geographical limitations, among others [3, 10]. For diabetes medications (oral and insulin), it was found that the main means of obtaining them in the North was private pharmacies, suggesting regional disparities in access.

The problems with access to medicines were also reported in the National Survey on Access, Use and Promotion of Rational Use of Medicines (PNAUM- Services) [27], which pointed to statistically significant differences in access to essential medicines among regions of the country, as well as according to type of medicine. A study carried out on the basis of the VIGITEL survey (2011) showed that it is precisely in the capitals of Brazilian regions with less economic development and a greater number of socially vulnerable people that patients most needed to make direct disbursements to access treatment for hypertension and diabetes [21]. This finding points out how unequal health care is in a country with continental dimensions such as Brazil.

This study highlights the difficulties of obtaining medication in the North region, which clearly demonstrates geographical inequalities in the field of health, when compared with the South region. Access to medicines in that region is a challenge for patients and for the management of health services; in addition, the medicines are financed per capita, which is a disadvantage for the North region, where costs are higher [23, 28].

The idea of inequality caused by geographical difficulty in accessing medicines is further strengthened by analysing some of the different spaces for health care and access to medication prescriptions, such as the Family Health Strategies (FHS) [29]. While the North region has family health coverage of more than 63.0%, the Southeast region has less than 54.0% [30]. This finding converges with the hypothesis that it is not enough to guarantee access to health, if the ability of users or patients to obtain them is not considered. Also, another point to be considered is the hypothesis that the distribution of health services is not proportional to the distribution of demands [29].

Major limitations of this study, since it contains self-reported information, are the memory bias of the interviewees, possible differences in the understanding of some issues and the selection bias, since the survey did not include individuals living in households without a landline. In addition, it should be noted that the results are valid and comparable only capitals.

## Conclusion

The results found in this population survey expand knowledge about access to medicines in capitals, and highlight the growth and expansion of pharmaceutical services, with free availability of medicines for diabetes and hypertension present in health centers and pharmacies affiliated with PFP. On the other hand, the results also point out that there is still a portion of the population without access to these essential medicines, especially in the most economically vulnerable regions. This result is evidence that there are regional disparities and, in this way, it contributes insights to the management of existing public health policies.

## Data Availability

All data are public and can be found on the official website of the study https://saude.gov.br/saude-de-a-z/vigitel
